# Fatal meningococcemia

**DOI:** 10.3402/jchimp.v1i4.11584

**Published:** 2012-01-26

**Authors:** John Tabacco, Elizabeth Suniega, Fardad Sarabchi, Dimitra Mitsani

**Affiliations:** Union Memorial Hospital, Baltimore, MD, USA

**Keywords:** Infectious disease

## Abstract

Within the past six years, a case of bothWaterhouse-Freidrichsen Syndrome and fulminant meningococcemia have presented to Union Memorial Hospital. Both cases presented in markedly different fashions, differed in microbiologic serogrouping, showed minimal histopathologic similarities; however achieved ultimately the same outcome through two different pathological pathways. The following case reports illustrate two mechanisms through which *N. Meningitis* may pathogenize a host, both leading to complete cardiovascular collapse in less than 12 hours.

‘No other infection so quickly slays.’ The truth behind this quote, which was found in Herrick's 1919 article entitled ‘Extrameningeal meningococcal infections,’ remains as valid today as it was when he stated it more than 90 years ago, as few other bacterial species (besides *Neisseria meningitidis*) have since been found to have the capacity to kill healthy patients in less than 24 hours (1). Although the descriptive history of meningococcal disease dates back to the 16th century, the historical origin of acute adrenal apoplexy, the so-called ‘Waterhouse-Friderichsen syndrome,’ (WFS) a rare form of acute, rapidly-progressing, fulminant meningococcemia with bilateral adrenal gland hemorrhage, lies in a pre-antibiotic and pre-technological era (2). During the late 19th century as microscopy, culturing techniques, and schemes for pathogen classification were initially being developed, medicine relied on relatively few diagnostic tools, was largely observational, and communication between most scientists was limited (3). It was during this time when reports of ‘catastrophic bacterial syndromes’ were first illustrated. Time has revealed many of these observations to be early clinical encounters with *Neisseria meningitidis*.

Arthur Voelcker reported the first description of acute adrenal apoplexy in a two-year-old English girl in 1894 (4). Subsequently, independent case reports with analogous observations were made between 1898 and 1901 by Garrod and Drysdale (5), Andrewes (6), Talbot (7), Blaker and Bailey (8), and Little (who was the first author to elaborate on a hypothesis that involved acute Addison's disease and the action of then-unknown cortisol) (9). Furthermore, two detailed articles about acute adrenal apoplexy in children appeared in *The Lancet*, authored by Langmead in 1904 (10) and Andrewes – the first person that was able to isolate *N. meningitidis* from the blood of an affected patient – (11) in 1906, prior to that which was written about a similar case report by Rupert Waterhouse in 1911 (12).

A change in the epidemiology of WFS, from a pediatric disease that was almost exclusively reported in English children to a worldwide disease that could affect patients of all ages, first began to occur around 1914 in World War I-related military training camps (1, 3). After many early authors had theorized about the etiology of suprarenal apoplexy – most commonly with relation to an elusive toxin or infection (especially smallpox) – McLagen and Cooke definitively determined *N. meningitidis* to be the causative organism of this disease in 1916 (13). Carl Friderichsen published an article in the Danish literature about two pediatric cases of acute adrenal apoplexy in 1917, and a review article encompassing information from 28 such cases in the German literature in 1918, and was the first author to emphasize the importance of adrenal cortical insufficiency in the pathogenesis of suprarenal apoplexy (14, 15). In 1933, ‘Waterhouse-Friderichsen syndrome’ was officially named (for currently unknown reasons) as a unique disease entity by Eduard Glanzmann, while he was giving a presentation on the subject (16). By the time that Friderichsen wrote his second review article about WFS in 1955 in the English literature, a wealth of information about its epidemiology, pathogenesis, diagnosis, and treatment (especially with respect to sulfonamides, penicillin G, chloramphenicol, and synthetic corticosteroids) had been discovered (17). Since the mid-1950's, further advances in knowledge about meningococcal disease and WFS have been achieved.

Interestingly, within the past six years, a case of both Waterhouse-Friderichsen syndrome and fulminant meningococcemia have presented to Union Memorial Hospital. Both cases presented in markedly different fashions, differed in microbiologic serogrouping, and showed minimal histopathologic similarities. However, they ultimately achieved the same outcome through two different pathological pathways. The following case reports illustrate two mechanisms through which *N. Meningitidis* may pathogenize a host, both leading to complete cardiovascular collapse in less than 12 hours.

## Case reports

### Case 1

A 46-year-old male bus driver with past medical history of obstructive sleep apnea presented to an emergency department in Baltimore Maryland in early July, 2011 at approximately 1:00 pm, complaining of sudden onset severe, left sided chest pain, shortness of breath, back pain, chills, and one episode of a non-witnessed seizure with loss of consciousness as he was preparing to depart on his bus route early in the morning. The patient denied any prodrome to presenting symptoms, stating he had felt fine the day prior. He denied sick contacts.

His temperature on admission was 101.1, blood pressure was 126/71 mmHg, and heart rate was 96 BPM. He was an obese, well developed male who was ill looking. He was alert and oriented with no nuchal rigidity. Lungs were clear and no cardiac murmurs were auscultated. No abdominal or flank tenderness was noted. No rashes, petechiae or purpura were noted. Neurological exam was non focal.

Significant laboratory values were: WBC: 3.8 k/UL, PMN: 90.9%, troponin: 0.057 ng/ml, Hgb: 12.8 gm/dL, PLT: 198K/UL, BUN: 12, Cr: 0.68, CK: 189. ECG showed non-specific T wave abnormalities.

Chest radiograph revealed questionable lower lobe infiltrate and piperacillin-tazobactam was started empirically. Chest CT was negative for pulmonary embolism.

At 7:45 pm, while in the ER, the patient began to hemodynamically decompensate as blood pressure dropped to 80/60 mmHg. The patient was given two liters of normal saline and was moved to the Intensive Care Unit. Cardiopulmonary arrest occurred at approximately 8:45 pm. Cardiopulmonary resuscitation was started and intubation was attempted but failed and pulse was unable to be established. Approximately eight hours after presenting to the Emergency Room the patient was pronounced dead.

At 8:00 am on the following morning, morphology of blood cultures returned as Gram negative diplococci, with speciation later to return as Neisseria meningitides serogroup Y.

**Fig. 1:**
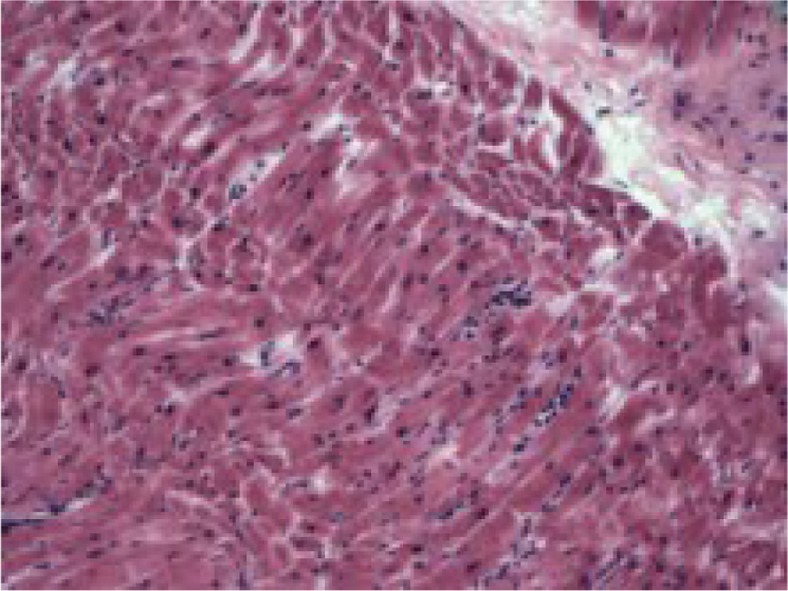
Myocarditis.

Later autopsy revealed myocarditis (Fig. 1) Final diagnosis was fulminant meningococcemia.

### Case 2

A 19-year-old male college student was brought into the emergency department by ambulance at approximately 1:00 am on 26 October 2005. The patient believed that he was having an ‘allergic reaction to Chinese food’ that he had consumed an hour prior to developing the presenting symptoms of hot flashes, shortness of breath, lightheadedness, generalized weakness, and myalgia.

On physical exam he was alert, pale, clammy, tachycardic, normotensive, and afebrile. There was no rash. The patient was treated initially for a presumed allergic reaction. On subsequent re-evaluation at 7:00 am and 8:00 am, he remained tachycardic with no improvement of symptoms. Initial laboratory workup was significant for a WBC: 2.2 k/UL, Hgb: 12.1 gr/dl, PLT: 35 K/UL, bandemia of 36%, non-anion gap metabolic acidosis (CO2: 16 mmol/L); Urine toxicology screen was negative.

Approximately five hours later, the patient developed restlessness, diaphoresis, and became disoriented. Blood cultures were sent and broad spectrum intravenous antibiotics were started. He was sent to the Intensive Care Unit. At 9:45 am he became hypotensive, failed to respond to fluid resuscitation and went into a state of pulseless electrical activity. Cardiopulmonary resuscitation continued for 45 min and patient was pronounced dead at 10:30 am, less than nine hours from time of presentation.

The following day, blood cultures grew gram negative diplococci later speciated as Neisseria meningitidis serogroup B. Review of school records showed that he had been vaccinated with the meningococcal vaccine.

**Fig. 2:**
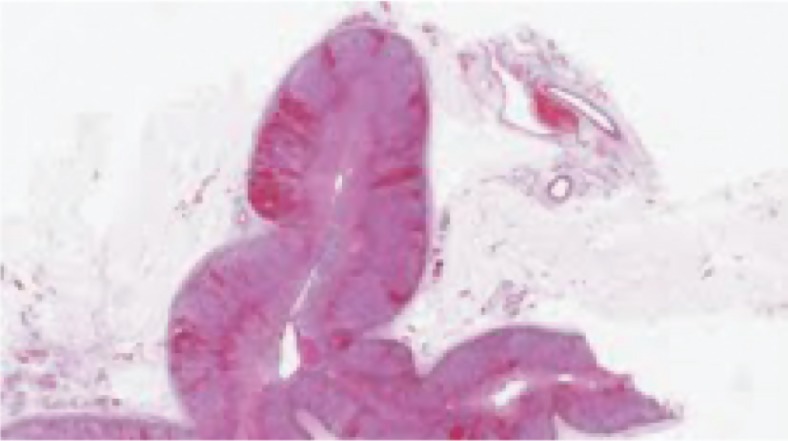
Adenal cortex hemorrhage.

On autopsy, there were macroscopic bilateral areas of adrenal hemorrhage consistent with Waterhouse-Friderichsen syndrome (Fig. 2) as well as florid conjunctival petechiae and bilateral subconjuntival hemorrhage. Myocarditis was noted.

## Discussion

*Neisseia meningitides* is a gram negative aerobic diplococcus that colonizes human nasopharynx in up to 10% of the population (18). Humans are the sole reservoir for this pathogen which is spread by physical contact or by inhalation of respiratory droplets (18). Infections in industrialized countries occur sporadically or in outbreaks, tend to exhibit seasonality (late winter – early spring) and are observed mainly in crowded settings such as schools or military camps where transmission is readily facilitated. There are 13 antigenically distinct serogroups of *N. meningitides*. Serogroups B, C and Y account for the majority of disease in the United States whose annual incidence amounts to 1,000 cases (19).

Neisseria meningitis is the second most common pathogen of community acquired meningitis (19) but meningococcal disease can present with extremely rapidly developing fatal outcomes in the absence of meningitis as in our two cases. Our second case is an example of Waterhouse-Friderichsen syndrome with extensive adrenal hemorrhages; a catastrophic development in a septic patient. Our first case had a similar clinical course but without the adrenal hemorrhage. This autopsy result surprised us, but serves to point out the unique virulence of this organism, as well as illustrating the important point that myocarditis during *N. Meningococcus* infections increases the mortality of this destructive pathogen.

Meningococcus possesses an array of characteristics that serve to evade the host immune response, rendering havoc in individual cases (20). First, structural components of its service display molecular mimicry of host cells (20). Second, the organism employs genetic transformation- reuptake of DNA from other bacteria in the environment along with iron scavenging and chelation to a binding human lactoferrin that selects for survival and commensal state and allows for rapid multiplication and outer membrane proteins which promote attachment and host invasion (21). The unique structure of the outer membrane liposaccharide leads to bleb formation and release of potent endotoxin that quickly achieves high blood stream levels readily culminating in clinical sepsis (21). Finally, the inner bacterial capsule is well known to display resistance to complement phagocyte mediated lysis (22).

One hundred years ago Waterhouse reported a disease that was humbling in its ferocity. A century has passed and our understanding is at a rewardable depth. We are equally impressed and humbled today.
